# A Rare Complication of Laparoscopic-Assisted Percutaneous Gastrostomy Tube Placement Through the Posterior Gastric Wall

**DOI:** 10.7759/cureus.78659

**Published:** 2025-02-06

**Authors:** Andrew J Landau, Brendan R Martino, Zachary Neubert

**Affiliations:** 1 Internal Medicine, Tripler Army Medical Center, Honolulu, USA; 2 Gastroenterology, Halifax Medical Center, Daytona Beach, USA; 3 Gastroenterology, Tripler Army Medical Center, Honolulu, USA

**Keywords:** eneteral nutrition, gastric tube, gastroenterology and endoscopy, gastrostomy tube complication, iatrogenic gastric perforation, laparoscopic gastrostomy, percutaneous gastrostomy, postop complication, post-stroke dysphagia, tube feeding

## Abstract

Complications of gastrostomy tube placement and enteral feeding are common. However, most are minor and without serious adverse effects, such as superficial skin infections, peristomal leakage, tube dislodgement, and minor bleeding. Misplacement of a gastrostomy tube through the anterior and posterior gastric walls is a rare but serious complication. If not detected and corrected, it could have serious and potentially life-threatening consequences for the patient. We present a case of a 78-year-old male with acute stroke and resultant severe oropharyngeal dysphagia requiring long-term enteric feeding tube placement complicated by a malpositioned gastrostomy tube that was placed through both the anterior and posterior walls of the stomach during a laparoscopic procedure, resulting in coffee-ground hematemesis on the first postoperative day. Investigation revealed the malpositioned tube, which was surgically repaired on time without significant morbidity.

## Introduction

Gastrostomy tube placement for enteral feeding, decompression, and medication administration is a common procedure, with over 250,000 tubes placed annually in the United States. The majority are inserted via percutaneous endoscopic gastrostomy (PEG), followed by radiologic percutaneous gastrostomy (RPG) [1-2]. Surgical tube placement, either laparoscopic-assisted percutaneous gastrostomy (LAPG) or open gastrostomy tube placement, is less common due to longer operative times, need for general anesthesia, and higher costs. Surgically placed tubes do have specific indications and offer improved techniques to bypass certain contraindications to PEG and RPG, such as avoiding intervening colon or other overlying organs that impede a direct path to the gastric body. It also allows for the surgeon to address adhesions and navigate anatomic changes resulting from previous abdominal surgery. Overall complication rates are similar among PEG, RPG, and laparoscopic methods, with each considered safe and effective. Major complication rates, including perforation, hemorrhage requiring transfusion, necrotizing infection, and 30-day mortality, range from 2% to 15% [3-5]. Ultimately, the choice of method and technique will depend on proper pre-procedural planning, availability of resources, well-trained proceduralists, and patient-specific factors.

We present the case of a 78-year-old male who developed coffee-ground hematemesis following the placement of a laparoscopic-assisted gastrostomy tube through both the anterior and posterior gastric walls for enteral nutrition access due to neurologic dysphagia. While this is a known complication, it is rarely described or discussed in the literature and is likely avoided or immediately recognized in most cases due to techniques aimed at prevention, detection, and confirmation of proper tube placement. It could result in catastrophic outcomes such as sepsis and peritonitis from feeding and/or medication administration through the tube if not recognized and addressed early. It is, therefore, an important complication for providers to be aware of and understand how to diagnose and address expediently.

## Case presentation

A 78-year-old male with a past medical history of hyperlipidemia, hypertensive heart disease with diastolic dysfunction, stage 3a chronic kidney disease, and dementia was admitted to an acute care hospital in Hawaii for acute ischemic stroke of the left basal ganglia and right mesial temporal lobe with symptoms including dysarthria, oropharyngeal dysphagia, and right hemiparesis. He was not a candidate for thrombolysis and was started on dual antiplatelet therapy with aspirin and clopidogrel. A clonidine patch was started for hypertension following 48 hours of permissive hypertension. The stroke was diagnosed with CT and MRI of the brain. A barium swallow study confirmed oropharyngeal dysphagia; the family and patient decided to proceed with gastrostomy tube placement for long-term enteral nutrition. An LAPG tube was placed on the fifth hospital day by the on-call general surgeon, utilizing a transabdominal introducer insertion technique. Three T-fasteners were placed for gastropexy, and the Seldinger technique was used to dilate the tract and insert a 14F balloon-retention gastrostomy tube through a peel-away introducer sheath. After securing the tube in position, saline was instilled through the gastrostomy tube (g-tube), and aspiration confirmed the presence of gastric contents. The patient was not immediately started on enteral feedings through the tube, but medications were administered four hours after tube placement.

**Figure 1:**
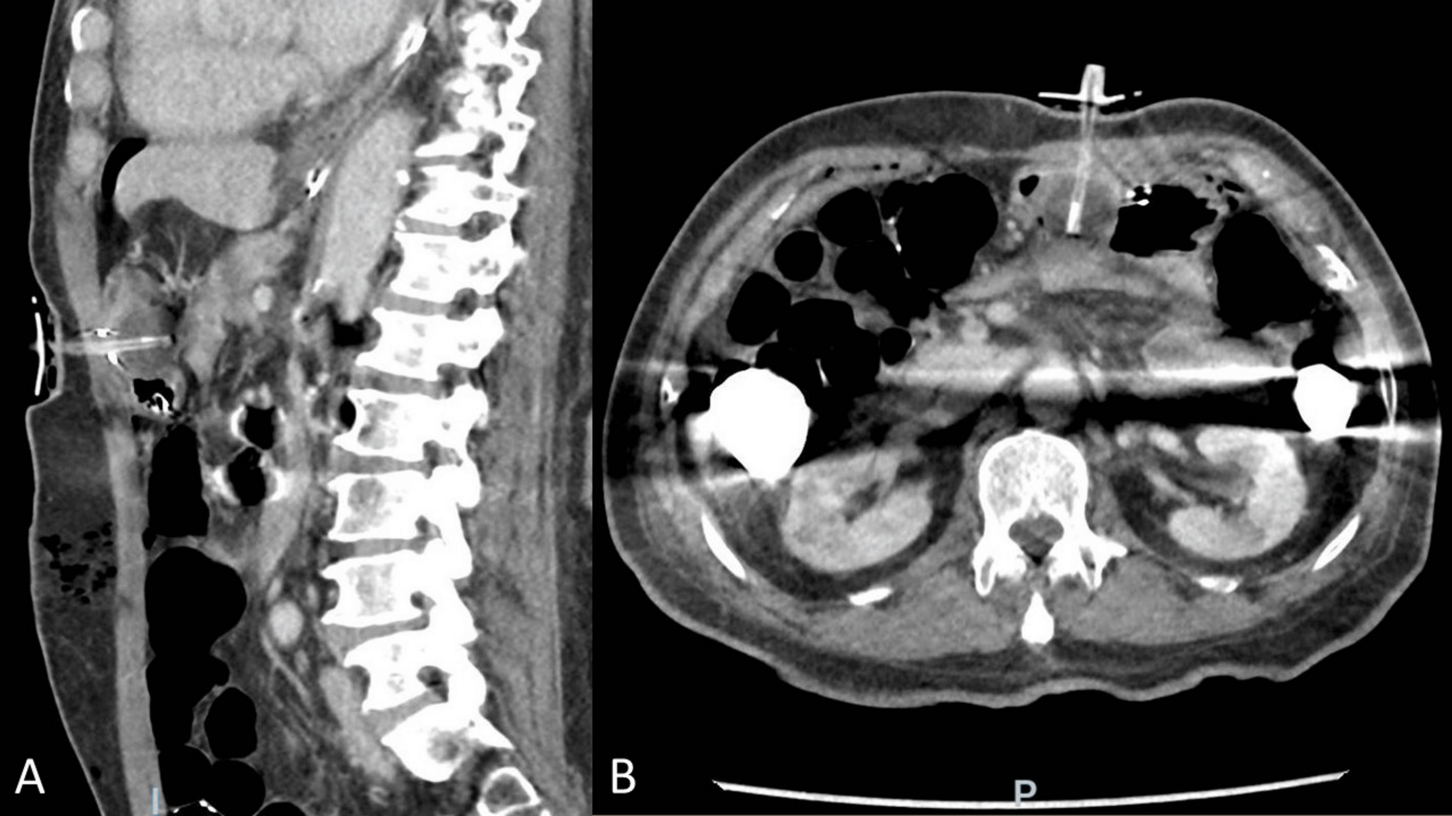
Computed tomography (A) sagittal and (B) transverse/axial images showing postoperative changes after laparoscopic gastrostomy tube placement through anterior and posterior walls of the stomach.

On the first postoperative day, the patient had three episodes of coffee-ground hematemesis overnight without hemodynamic instability and developed a leukocytosis of 18,000 per microliter. He was afebrile and tachycardic with a heart rate of 100-120 beats per minute. On physical exam, the surgical site was clean without external signs of hemorrhage or infection. The patient was tender around the laparotomy and g-tube sites without signs of distention or peritonitis. Due to gastrointestinal (GI) bleeding and leukocytosis, an abdominal CT scan without contrast was performed, revealing numerous small foci of intraperitoneal air and surgical changes in the periumbilical region with the stomach approximating the anterior abdominal wall, thought to be consistent with the laparoscopic intervention. The gastrostomy tube appeared to terminate in the distal gastric body. It also showed complete apposition of the anterior and posterior gastric walls at the gastrostomy tube site, with the stomach appearing compressed between the balloon and the abdominal wall (Figure 1). The latter findings were not immediately recognized, and gastroenterology was consulted for endoscopic evaluation and treatment of the hematemesis.

**Figure 2:**
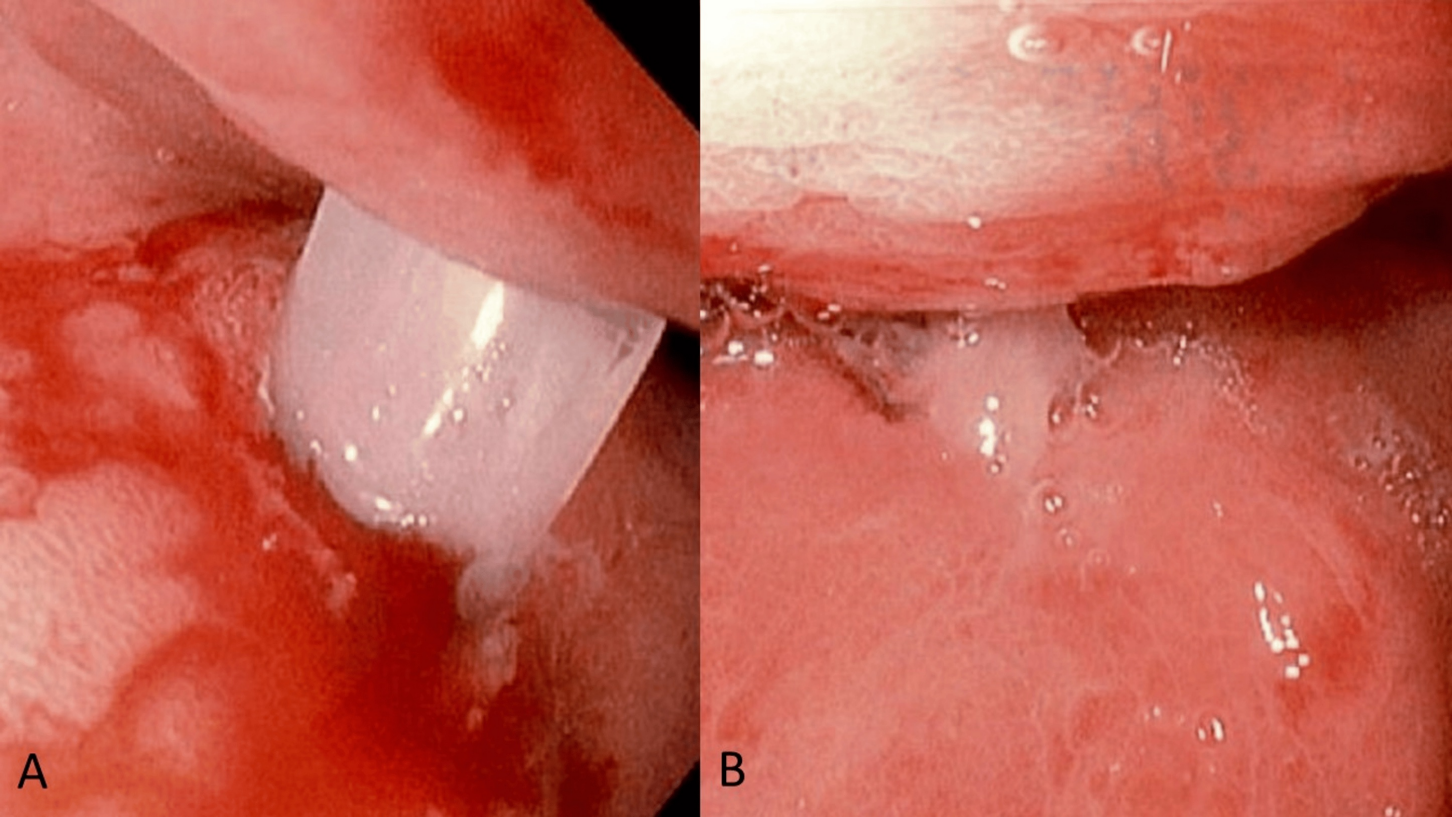
(A-B) Esophagogastroduodenoscopy findings showing a malpositioned gastrostomy tube entering the anterior gastric wall and exiting the posterior gastric wall, with clean-based peritubular ulcers on the gastric mucosa of both walls.

**Figure 3:**
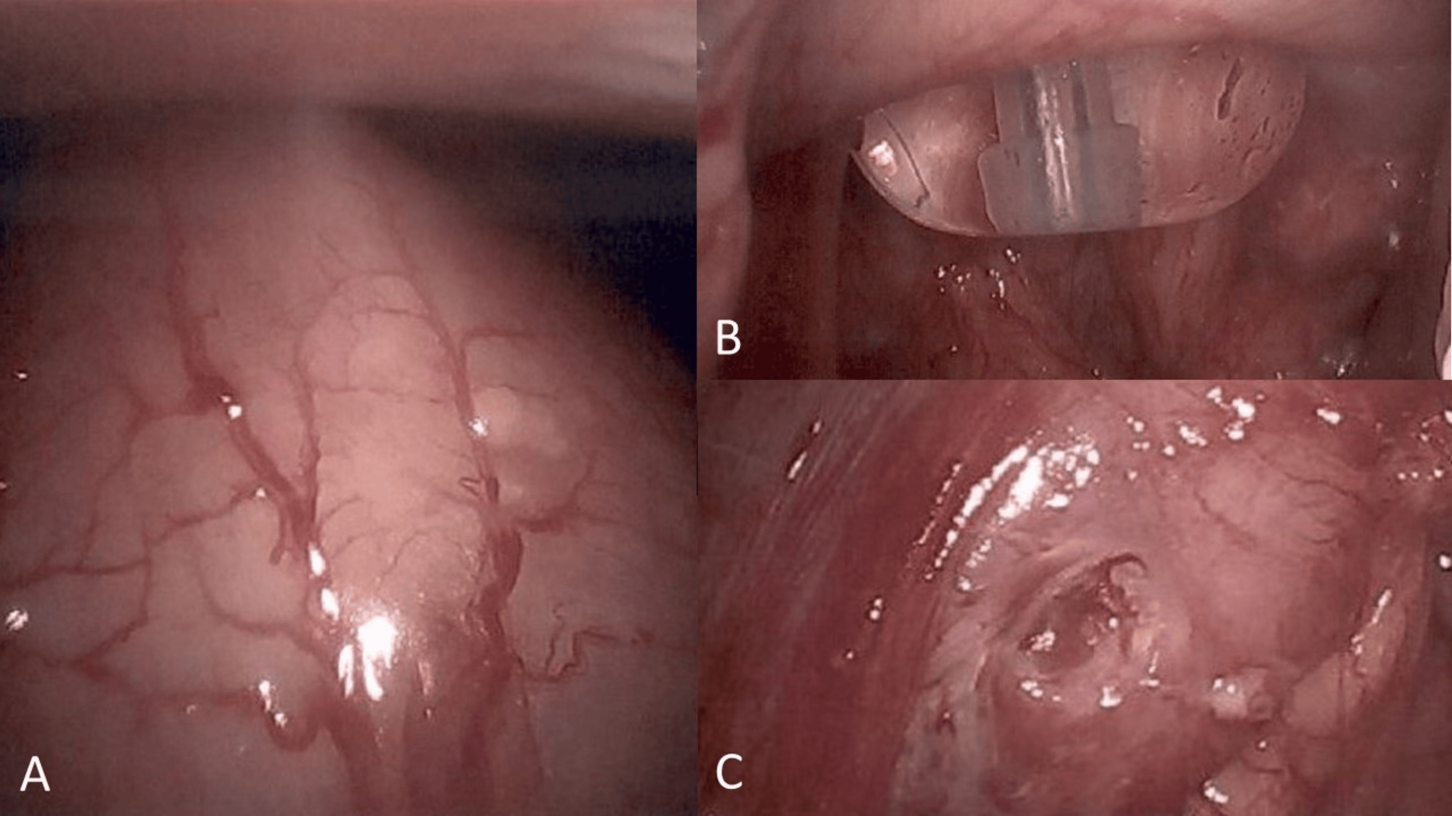
Intraoperative images of (A) the stomach, (B) a malpositioned gastrostomy tube passing through the posterior gastric wall, and (C) a posterior wall mucosal defect after tube removal.

**Figure 4:**
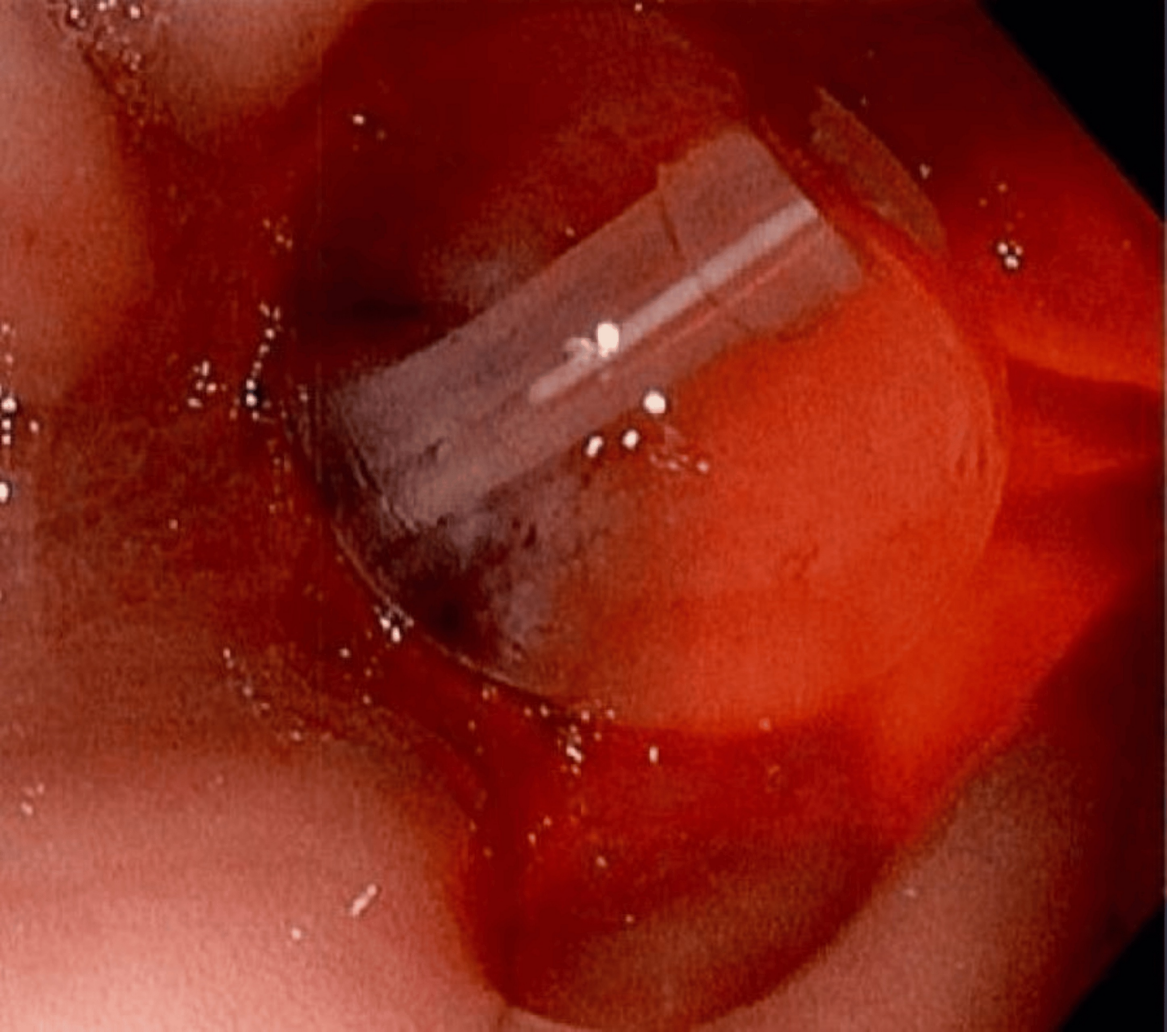
Esophagogastroduodenoscopy image confirming proper intragastric placement of the gastrostomy tube after repositioning.

Esophagogastroduodenoscopy (EGD) performed on postoperative day 3 demonstrated hyperemic mucosa throughout the stomach, with a gastric tube seen entering the anterior gastric body and exiting through the posterior gastric wall, as well as clean-based ulcers around the entrance and exit sites (Figure 2). This was presumed to be the cause of GI hemorrhage. The patient was put on pantoprazole 40 mg intravenously (IV) twice daily for three days, then transitioned to once-daily dosing. The surgeon was contacted for surgical management of the mispositioned tube and posterior wall gastrostomy site. Diagnostic laparoscopy was performed the same day without evidence of intra-abdominal contamination and laparoscopic manipulation/rotation of the stomach revealed the gastric tube exiting the posterior gastric wall (Figure 3). The tube was repositioned intragastrically by pulling on the tube percutaneously and an endoscopic GI anastomosis stapler was used to close the defect from the serosa. Tube placement was confirmed with injection and aspiration of saline and by intraoperative EGD (Figure 4), and the abdomen was closed. The patient tolerated tube feeds without complications and was discharged two days later.

## Discussion

Enteral feeding tube placement can be performed endoscopically, radiologically, or surgically in patients with a functional GI tract who are unable to achieve adequate caloric intake. The primary goals are to improve nutrition, reduce mortality, and enhance quality of life. Percutaneous methods are generally preferred, with PEG being most commonly utilized, though RPG is gaining in popularity. Surgically placed gastrostomy tubes are typically reserved for cases in which there is a contraindication to PEG/RPG, or the patient is already having surgery for another reason. Various techniques may be used for placement with each method and are summarized in Table 1, along with their specific indications and contraindications. The selection of method, technique, and timing of intervention should carefully consider facility resources, provider expertise, and patient-specific factors. A thorough discussion of potential complications and realistic expectations of benefit should take place with the patient and family before proceeding with tube placement. Common complications and manners of minimizing their risks have been extensively reported in the literature and various society guidelines. We will focus on specific topics relevant to this case.

**Table 1:** Percutaneous gastrostomy tube insertion techniques. GI, gastrointestinal, EGD, esophagogastroduodenoscopy

Tube insertion techniques	
Endoscopic gastrostomy tube (PEG)	
Pull (Ponsky-Gauderer) Technique	After endoscopic insufflation of stomach and confirmation of safe tract, guidewire is inserted transabdominally and pulled by endoscopic snare out of oropharynx, tube is attached and pulled through the abdominal wall by the attached guidewire while under direct endoscopic visualization.
Push (Sachs-Vine) Technique	After endoscopic insufflation of the stomach and confirmation of a safe tract, the guidewire is inserted transabdominally and pulled out of the oropharynx using an endoscopic snare. The tube is then pushed over the guidewire through the abdominal wall under direct endoscopic visualization.
Introducer (Russel) Technique	After endoscopic insufflation of the stomach and confirmation of a safe tract, gastropexy is performed with T-fasteners. The Seldinger technique is then used to dilate the external tract over a guidewire, followed by the insertion of a peel-away introducer sheath. The tube is subsequently fed into the introducer and advanced through the abdominal wall under direct endoscopic visualization.
Indications	Enteral feeding access or GI decompression is the preferred method for most patients.
Contraindications	Pharyngeal or esophageal obstruction, coagulopathy, transverse colon or another organ overlying the stomach body, inability to confirm a safe tract with transillumination and finger palpation, high aspiration risk, large varices, upper GI bleeding risk, or other contraindications to EGD.
Radiologic Gastrostomy Tube (RPG)	
Introducer Technique	Under fluoroscopic guidance, nasogastric tube insufflation of the stomach, gastropexy, and then tube insertion via introducer technique. Placement confirmed with fluoroscopy.
Pull Technique	Under fluoroscopic guidance, nasogastric tube insufflation of the stomach, gastropexy, and then tube insertion via pull technique. Placement confirmed with fluoroscopy.
Indications	Enteral feeding access or GI decompression, unsuccessful PEG or contraindications to PEG or EGD, especially pharyngeal/esophageal obstruction/cancer, high risk of aspiration or upper airway or GI bleeding with EGD.
Contraindications	Contrast allergy, coagulopathy, transverse colon or another organ overlying the stomach body, and inability to achieve safe access to the stomach without damaging surrounding structures. The pull technique shares similar contraindications with PEG.
Laparoscopic-assisted percutaneous gastrostomy tube (LAPG)	
Introducer Technique	Insertion of laparoscopic trocars (2 or 3) and then insertion via the introducer technique.
PEG Technique (LAPEG)	Combination of LAPG with PEG insertion.
Indications	Enteral feeding access or GI decompression, unsuccessful PEG/RPG or contraindications to PEG/RPG, anatomic anomalies making PEG/RPG unsafe, previous complex upper abdominal surgery or extensive adhesions, and other need for surgery at the time of placement.
Contraindications	Contraindications to general anesthesia or abdominal surgery.

Posterior gastric wall perforation during gastrostomy tube placement is a known theoretical complication; however, to our knowledge, it has not been previously described and is rarely discussed as a complication in recent literature. It is rare when performed by an experienced surgeon and with proper patient selection. Inadvertent perforation of structures anterior to the stomach such as the colon, small bowel, and liver are complications that are more easily avoided by LAPG due to direct visualization of the intraperitoneal cavity and structures surrounding the stomach. Posterior gastric wall perforation during needle insertion for T-fastener placement or initial access into the stomach has been reported, but it rarely leads to the placement of a tube through the posterior wall. This may be due to decreased utilization of surgical methods and the capability for direct intragastric and fluoroscopic visualization during PEG and RPG, respectively. However, it is also rare in surgical placement, and evolving techniques across all methods aimed at the prevention and early detection of complications likely result in very few cases progressing to prolonged tube placement through both gastric walls, among other technical failures. Proper insufflation of the stomach to ensure separation of the anterior and posterior gastric walls is a universally applied preventive measure, regardless of the method used. Endoscopic insufflation is achieved during PEG, while a nasogastric tube is often used to inflate the stomach in RPG. LAPG may utilize either a nasogastric tube or insufflation through the abdominal wall after needle insertion and before dilator placement. It must be considered that abdominal insufflation to a pressure of 15 mmHg is often utilized in laparoscopy and may increase the external pressure on the stomach, causing an increased risk of approximation of anterior and posterior gastric walls. The introducer method, in which the Seldinger technique is used to insert the tube through a peel-away sheath, as employed in this case, appears to be more prone to this complication. This is commonly used with LAPG and RPG. The RPG introducer technique allows for fluoroscopic visualization, and contrast extravasation from the stomach would presumably detect most cases of posterior wall placement. With LAPG, flushing gastric contents is one method to confirm placement; however, it may lead to false positives, as seen in this case. Visual confirmation of tube placement within the stomach, rather than through the posterior wall, would require dissection into the lesser sac. However, this is not practical for routine use due to the risk to surrounding structures and the low likelihood of this complication. If malposition is a concern, an intraoperative fluoroscopic or endoscopic study, or a radiographic tube study, where water-soluble contrast is injected into the tube followed by an abdominal radiograph, may help confirm proper placement. Postoperatively, endoscopy could also be considered. Our patient did not receive gastric tube feeds before developing coffee-ground hematemesis and was only administered medications through his tube, which was extremely fortunate. Had he received tube feeds or not exhibited hematemesis to alert the provider, he likely would have experienced more severe morbidity, such as intraperitoneal contamination, infection, and peritonitis, potentially requiring more complex surgical interventions (e.g., exploratory laparotomy with multiple washouts) and even posing a risk of death.

Neurologic dysphagia is a common manifestation of stroke occurring in up to 45% of hospitalized patients [6]. As recovery from stroke is variable and dependent on the location and extent of involvement of the central nervous system, gastrostomy tube placement is often indicated in these patients to improve nutrition and long-term outcomes. In general, gastrostomy tubes should be considered for patients who are expected to have a meaningful recovery and require enteral nutrition support for more than four weeks. Investigations have shown worse outcomes among patients undergoing gastrostomy tube placement during acute hospitalization after stroke and reduced complication rates when placed in an elective setting at least four weeks after the initial event [7-13]. As such, some experts recommend using nasogastric tube feeding for at least 24 hours up to three to four weeks, after which a gastrostomy tube should be placed if the need for enteral access persists [7-15].

Ultimately, the most serious potential consequences of this complication were avoided due to astute postoperative care and close observation. A combination of vital signs, laboratory data, and physical exam findings led the clinician to appropriately obtain imaging and consultation for upper endoscopy so that the complication could be identified and corrected early before causing more serious harm to the patient. It is especially important to monitor patients closely during the early postoperative and feeding periods to detect and address complications promptly, minimizing adverse consequences.

## Conclusions

This case presents a 78-year-old male who developed coffee-ground hematemesis, leukocytosis, and eventually peritonitis due to LAPG tube placement through both the anterior and posterior walls of the stomach, which could have been a catastrophic complication if not detected and managed quickly. A variety of patient and facility factors should be considered when determining the need for surgical feeding tube placement. Prompt recognition of this serious complication by the surgeon is crucial in preventing morbidity and mortality. This case highlights that malpositioning complications can present subtly and may not become apparent until several days after the procedure. Clinicians must ensure there is no doubt regarding the proper placement of the PEG tube before initiating tube feeding. If any uncertainty exists, verification should be performed using contrast-assisted radiographic studies or endoscopic evaluation. Through this case, we highlight the importance of proper patient selection, method and technique choice, timing, and risk factor optimization. In addition, we discuss the relative limitations of various gastrostomy tube placement methods.
